# Measurement of Redox Potential in Nanoecotoxicological Investigations

**DOI:** 10.1155/2012/270651

**Published:** 2011-10-31

**Authors:** Ratna Tantra, Alex Cackett, Roger Peck, Dipak Gohil, Jacqueline Snowden

**Affiliations:** ^1^Nanoanalysis Group, National Physical Laboratory, Hampton Road, Teddington, Middlesex TW11 0LW, UK; ^2^Advanced Engineered Materials Group, National Physical Laboratory, Hampton Road, Teddington, Middlesex TW11 0LW, UK

## Abstract

Redox potential has been identified by the Organisation for Economic Co-operation and Development (OECD) as one of the parameters that should be investigated for the testing of manufactured nanomaterials. There is still some ambiguity concerning this parameter, i.e., as to what and how to measure, particularly when in a nanoecotoxicological context. In this study the redox potentials of six nanomaterials (either zinc oxide (ZnO) or cerium oxide (CeO_2_)) dispersions were measured using an oxidation-reduction potential (ORP) electrode probe. The particles under testing differed in terms of their particle size and dispersion stability in deionised water and in various ecotox media. The ORP values of the various dispersions and how they fluctuate relative to each other are discussed. Results show that the ORP values are mainly governed by the type of liquid media employed, with little contributions from the nanoparticles. Seawater was shown to have reduced the ORP value, which was attributed to an increase in the concentration of reducing agents such as sulphites or the reduction of dissolved oxygen concentration. The lack of redox potential value contribution from the particles themselves is thought to be due to insufficient interaction of the particles at the Pt electrode of the ORP probe.

## 1. Introduction

The size of engineered nanomaterials makes many novel and innovative products, as evident by the increasing number of commercially available nanotechnology products. Thus, there is a huge concern surrounding the potential toxicity of these nanomaterials and there is a need to sufficiently test such materials. The goal here is to understand and control risk, and both toxicity testing and physicochemical characterisation should be conducted. Although our current understanding of risk associated with nanomaterials is limited, attempts have been made in order to assess this systematically. Recently, Aschberger et al. [1] have carried out a risk assessment based on several case studies. They have indicated the risk expected from metal and metal oxide nanomaterials, which was particularly relevant in the case of algae and Daphnia. They have attributed the risk from such materials their exposure to both the particles and corresponding dissolved ions. 

There is a general consensus within the nanoecotoxicological community that physicochemical characterisation of nanomaterials in complex media is not a trivial matter, and so the reliability of such measurements is vital if we are to understand and control the risk imposed by nanomaterials [2, 3]. In recent years, the OECD initiative has adopted a holistic approach to this problem and that physicochemical characterisation should be carried out with as many parameters as possible, to include redox potential. The PROSPEcT (Ecotoxicology Test Protocols for Representative Nanomaterials in Support of the OECD Sponsorship Programme) project is the UK's contribution to this OECD initiative, and the UK is responsible for two types of nanomaterials: cerium oxide (CeO_2_) and zinc oxide (ZnO) [4]. Out of all seventeen OECD parameters identified, redox potential is the most ambiguous in its definition, what this parameter means and how it is measured, in a nanoecotoxicological context. 

Integral to any ecotoxicological investigation is the ability to measure the redox conditions of a given system as indicated by the redox potential. In nature, redox reactions are an important part of phenomena such as mineral weathering, bacterial respiration, and degradation of pollutants [5]. In soil chemistry, for example, the redox potential value can estimate whether the soil is aerobic or anaerobic, and whether chemical compounds such as Fe oxides or nitrate have been chemically reduced or are present in their oxidised form. In natural waters, redox reactions include the oxidation of organic matter and various reduction reactions such as the reduction of oxygen to water, nitrate to elementary nitrogen dioxide, iron (III) to Fe (II), sulphate to sulphide, and carbon dioxide to methane [6]. In terms of nanomaterial toxicity, redox potential is a parameter that has been associated with inducing oxidative stress. Recently, Burello and Worth [7], in their prediction of a given nanomaterial to induce oxidative stress, have developed a theoretical framework that combines measurements of nanoparticle particle size and redox potential. The need to accurately measure redox potential is evident, in particular we need to understand the extent that nanomaterials can influence the natural redox phenomena should these materials be released into the environment.

Redox potential is a measure of a system's affinity for electrons, and the measurement of redox potential will only have meaning when there are reduced and oxidised species, called the redox couple, in the liquid media. The redox couple undergoes a redox reaction, in which the reduction (gain of electrons) of one redox species is accompanied by the oxidation (loss of electrons) of another [6]. The movement of electrons, governed by kinetics (e.g., transport limitations of the redox species to the electrode), creates an electric potential. The potential measured is determined by the ratio of activities of oxidised and reduced species, as defined by the Nernst equation; this is a thermodynamic property [8]. The redox potential can be directly measured using a potentiometer (high impedance voltmeter) with an oxidation-reduction potential (ORP) electrode [9]. This is the recommended technique under the current OECD guidelines for the testing of nanomaterials (NMs) [10]; essentially it is a measurement of potential difference (in mV) across a two-electrode system, that is, an inert platinum (Pt) electrode and silver-silver chloride (Ag/AgCl) reference electrode. Rogers et al. have recently adopted this approach to measure the redox potential of nanomaterial dispersions, that is, cerium oxide dispersed in synthetic freshwater algal medium [11]. 

There are theoretical and practical difficulties associated with the measurement of redox potential and these, although not well recognised, have already been discussed for many years [6]. Firstly, redox potential is based on the concepts of equilibrium thermodynamics, and as such it can only be adequately measured at equilibrium. A reliable redox potential measurement requires that equilibrium be established not only at the electrode, but also among the various redox couples in solution. Many redox reactions are slow and often are at nonequilibrium conditions. Secondly, most redox potential measurements represent mixed potentials, and certain redox species may not contribute significantly towards the redox potential value; that is, not all will react sufficiently fast enough at the electrode and therefore will not contribute towards stable and reliable redox potential measurements [12]. If the particles themselves act as a redox species, then there are various factors that may prevent them contributing towards the final ORP value, including sedimentation events, diffusion limitations, and the barrier of electron exchange at the Pt electrode. If this is true, then redox potentials of nanomaterial dispersions are likely to be solely dominated by dissolved redox species in the media, rather than contributions arising from the particles themselves. In this study, we aim to investigate if this is the case. Although the ORP probe has been conveniently used in the past by scientists to directly measure redox potential, it is essential that the reliability of such data should be questioned when measuring nanomaterial dispersions. There is the risk that researchers may treat such a tool as a *black box* and thus may not be fully aware of the inherent limitations in the use of such a tool in these measurements. 

In this study, the ORP values of six nanomaterial (either ZnO or CeO_2_ dispersed in one of the four liquid media) dispersions will be measured using an ORP probe. Dispersions will be carried out according to the dispersion protocol as recommended under PROSPEcT. The ORP values of the nanomaterial dispersions will be compared relative to each other and to the corresponding media blank, to identify if there is any evidence of redox contributions as a result of the particles themselves. If there are redox contributions from the particles themselves, then this is likely to happen when dispersions are stable, as this would allow sufficient time for the particles to interact with the Pt electrode. Consequently as part of the investigation, the properties associated with the different nanomaterials will be characterised, parameters of interest to include particle size, zetapotential, and dispersion stability (as reported by the so-called *half life *values). Zeta-potential is a well-known parameter that characterizes the electric properties of solid surface in contact with liquid and is a way to probe surface charge. The magnitude of this value is related to dispersion stability, that is, the higher the value, the better the dispersion stability [13]. The concept of “half-life” has been put forward in the OECD guidelines as the measurand to indicate dispersion stability through time that is, the larger the half-life value; the longer it takes for the concentration to reduce by half and thus the more stable the dispersion. Lastly, the corresponding scanning electron microscopy (SEM) data, that is, the primary particle size (mean Feret diameter) and the corresponding standard deviation, will also be reported.

## 2. Experimental Section

All experiments were performed in a temperature-controlled laboratory, and for the redox potential measurements the temperature of the dispersions were monitored using a temperature probe (reported value of ~20°C) to ensure that any change in the readings was not attributed to temperature changes in the dispersions. 

### 2.1. Materials

The NMs supplied from the PROSPEcT programme were of two types, either CeO_2_ or ZnO, and are as follows:

- Nanograin CeO_2_ (from Umicore Belgium),
- Nanosun ZnO (from Micronisers, Australia),
- Micron ZnO (from Sigma Aldrich, UK),
- Z-COTE ZnO (from BASF, Germany),
- Micron CeO_2_ (from Sigma Aldrich, UK),
- Ceria dry CeO_2_ (from Antaria, Australia).

The particles were used as received and did not contain any added surface stabilisers.

DI water (resistivity of 18 Mohm) from a Millipore, MilliQ system was used to prepare all aqueous solutions and suspensions. 

For the purpose of zetapotential measurements, DI water with 5 mM sodium chloride (Sigma Aldrich, UK) was employed in addition to deionised water; the NaCl here served as background electrolyte for the measurement of zetapotential. The “recipes” (chemical compositions) used for making up the ecotox media were obtained from the University of Exeter, one of our collaborators in the PROSPEcT project. 

Three types of ecotox relevant media were prepared accordingly and for long-term storage, the ecotox solutions were autoclaved and kept refrigerated until needed.

- Seawater, in which 25 g per L of Tropic Marine Sea Salt (Tropical and Marine Limited) was made up, resulting in pH ~8.8.
- Daphnia freshwater media. This was prepared by firstly dissolving appropriate salts (196 mg CaCl_2_·2H_2_O, 82 mg MgSO_4_·7H_2_O, 65 mg NaHCO_3_, 0.002 mg Na_2_SeO_3_, as obtained by appropriate dilutions of a 2 mg/mL stock solution) in 1 L of DI water. Upon continued stirring, further DI water was added so that conductivity of the solution was between ~360 and 480 *μ*S/cm. End volume ~1–1.5 L. Final pH ~7.9.
- Fish freshwater media. This was prepared in three separate steps. First, salts (11.76 g CaCl_2_·2H2O, 4.93 g MgSO_4_·7H_2_O, 2.59 g NaHCO_3_, 0.23 g KCl) were dissolved separately in 1 L of DI water to make four separate stock solutions. Second, 25 mL of each salt stock solution was aliquot into a clean bottle and diluted in DI water (made up to 1 L volume). Third, 200 mL of the stock solution from step 2 was aliquoted and further diluted with DI water (made up to 1 L volume). Final pH ~7.3.

Nanomaterials were dispersed using the protocol as previously reported (Tantra, Jing, Gohil 2010) (http://www.nanotechia-prospect.org/publications/basic). Briefly, this involved weighing the nanoparticle powder into small, clean vials using an analytical mass balance. Dispersion was carried out by adding the appropriate liquid media (fish, daphnia, seawater, or DI water) dropwise (5 drops from a Pasteur pipette) and mixing using a spatula so as to produce a thick paste before adding 15 mL of liquid media and stirring gently, using the same spatula. The formation of a thick paste as a first step was necessary to allow the efficient displacement of powder-air interface with the powder-liquid interface. The subsequent deagglomeration step was carried out using an ultrasonic probe (130 Watt Ultrasonic Processors); this was done by inserting the ultrasonic probe tip (6 mm Ti) half way down the 15 mL volume of dispersed nanoparticles, and sonication was carried out with 90% amplitude for 20 s. After sonication, the nanoparticle suspension was diluted using the appropriate liquid media, in order to make up to 1 L total volume. A glass rod was used to gently mix the final dispersion, to ensure homogeneity. The dispersions (in the four different media) were stored in separate precleaned 1 L media bottles and left undisturbed. Dispersion concentrations were 50 mg/L. Analyses of redox potentials, half-lives, and zetapotentials were conducted on the day immediately after the dispersions were made. 

### 2.2. High-Resolution SEM

SEM images were obtained using a Supra 40 field emission scanning electron microscope from Carl Zeiss (Welwyn Garden City, Hertfordshire, UK), in which the optimal spatial resolution of the microscope is a few nanometres. In-lens detector images were acquired at an accelerating voltage of 15 kV, a working distance of *≈*3 mm, and a tilt angle 0°. The SEM was calibrated using a SIRA grid calibration set (SIRA, Chislehurst, Kent, UK). These are metal replicas of cross-ruled gratings of area 60 mm^2^ with 19.7 lines/mm for low magnification and 2160 lines/mm for high magnification calibrations, accurate to 0.2%. For analysis of the “as received” nanoparticle powders, a sample of each powder was sprinkled over a SEM carbon adhesive disc; one side of the carbon disc was placed securely on a metal stub, whilst the other side was exposed to the nanoparticle powder. Excess powder was removed by gently tapping the stub on its side until a light coating of powder on the surface became apparent. An adequate magnification was chosen for image acquisition for example, for the estimation of primary particle mean diameter, the shape and limits of the primary particles should become apparent. SEM micrographs were analysed by manually tracing contours of primary particles onto a transparency sheet. The transparency sheet was scanned for further image analysis using Image J software, which automatically calculated particle diameter dimensions. 

### 2.3. Redox Potential Measurements

Redox potentials were measured using an ORP Oakton Waterproof ORP Testr, purchased from Cole-Parmer, UK. This, in effect, measures the potential difference across two electrodes (a Pt electrode against a double junction Ag/AgCl reference electrode). The ORP instrument manufacturer has specified a resolution of ±1 mV, with an accuracy of ±2 mV.

The electrode was used in accordance with the manufacturer's instructions. Prior to use, the electrode was preconditioned in clean tap water for 30 minutes before a final rinse with distilled water. When making measurements, the electrode was carefully placed in a vial containing the nanomaterial dispersion sample; there must be sufficient liquid sample to cover the sensing element. The electrode was carefully stirred a little and then placed in a fixed position, slightly above the bottom of the container. The signal output was allowed to settle for 5 minutes before a reading, the “field potential,” was noted. At this point, the signal was stable and there was no further change observed within the next few minutes. After measurement, the electrode was cleaned with tap water and rinsed with distilled water, after which further measurements could be made. When not in use, the electrode was stored in Oakton electrode storage solution. 

The redox potential ORP electrode was calibrated against YSI Zobell ORP Calibration Solution (purchased from Cole-Palmer). This reagent was made available in dry form and was reconstituted with 125 mL of DI water prior to use, after which the solution has ~6-month expiry date. This standard solution was used to verify the performance of the electrode at the beginning and end of the study. For Ag/AgCl reference, the redox potential value for Zobell solution was quoted to be 231 ± 10 mV (depending on temperature); at ~20°C, this value was ~237 mV. 

Redox potential measurements were carried out on freshly dispersed nanomaterial in the four chosen media, as detailed above. All field potential values recorded were subjected to an additive correction factor of +206 mV. This was necessary so that the final value was reported as if the reference electrode was a standard hydrogen reference electrode (SHE) instead of the Ag/AgCl, as previously documented [9]. The conversion from Ag/AgCl to SHE is typically on the order of 200 to 220 mV, and voltage correction is temperature dependent and also varies slightly with the concentration of KCl (~3.5 M) in the electrode filling solution.

### 2.4. Measurement of Half-Lives through Turbidity Measurements

Turbidity was measured using an HF Scientific-Micro100 RI turbidity meter (Cole-Palmer, UK); this meter has an infrared light source that meets the international standard ISO 7027 for turbidity measurements. The meter was calibrated on standards, which are based on AMCO-AEPA-1 microspheres; these standards are traceable to standard formazin suspension. Standard values of 1000, 10 and 0.02 NTU were used to calibrate the meter. Prior to use, the meter was allowed to warm up for 30 minutes. Sample cuvettes (HF Scientific (USA)) were used to hold the samples. Note that glass thickness may vary from cuvette to cuvette and within the same cuvette. Hence, individual vials were indexed; indexing of the cuvette entails finding the point of the cuvette that light passes through that gives the lowest reading and, once indexed, the holder can be marked accordingly. Prior to their use, cuvettes were cleaned, in accordance with manufacturer's instructions. This involved washing the interior and exterior of the cuvette with a detergent (2% Hellmanex in DI water); it was then rinsed several times in distilled water before finally rinsing in DI water. The cuvette was further rinsed with the sample two times before filling (30 mL) and analysed. The cuvette was placed into the meter and signal allowed to settle before taking readings. Turbidity readings were taken at regular time intervals. When not in use, the vials (containing the dispersions) were stored in the dark.

### 2.5. Zetapotential Measurements

Electrophoretic measurements were obtained using a Zetasizer Nano ZS (Malvern Instruments, UK) equipped with a 633 nm laser. The reference standard (DTS1230, zetapotential standard from Malvern) was used to qualify the performance of the instrument. Sample preparation involved filling a disposable capillary cell (DTS1060, Malvern). Prior to their use, these cells were thoroughly cleaned with ethanol and deionised water, as recommended by the instrument vendor. For analysis, the individual cell was filled with the appropriate sample and flushed before refilling; measurement was carried out on the second filling. Malvern Instrument's Dispersion Technology software (Version 4.0) was used for data analysis, and zetapotential values were estimated from the measured electrophoretic mobility data using the Smoluchowski equation, as documented in a previous publication [13]. 

## 3. Results and Discussion

Table 1 shows the redox potential results associated with dispersions of the PROSPEcT nanomaterials in four different media. The table also contains the redox potential values of the corresponding blank media, that is, liquid media with no nanomaterial. The values in brackets show the difference of ORP readings upon addition of the nanomaterials with respect to the corresponding blank media. 

Results show that ORP values are dominated by the type of liquid media used for dispersions. Nanomaterial dispersions in seawater resulted in a much smaller ORP values in comparison with dispersions made in other media. In addition, this is also the case with the ORP values of the corresponding blank media, that is, blank seawater having the smallest ORP value, of 384 mV, compared to the rest of the blank liquid media (redox potential values all above 400 mV). The ORP values reported here is not specific to a single chemical species and thus represent an aggregate oxidization-reduction of all species that can react at the Pt working electrode [14]. The fact that the type of liquid media itself seems to have some contribution to the final ORP values is not surprising, as dissolved redox species in the liquid can easily interact with the Pt electrode of the ORP probe. In fact, such ORP measurements are often employed as an accurate gauge of water quality and for the monitoring of dissolved species in the water [14]. Seawater in particular is shown to be more reducing in nature, that is, due to higher concentration of reducing agents (such as NO_2_^−^) in such media, if compared to the other liquid media. The presence of reducing agent has the effect of lowering the ORP value [15]. Furthermore, we expect a much-reduced level of oxidising agent such as dissolved oxygen in such a high saline solution, as the more saline the water can be the less oxygen the water can hold. If there is a reduction in oxidising agents, then this also has an effect of lowering the ORP value [16]. 

The ORP readings reported here were taken three times with very little variation among the replicates, that is, not more than ±2 mV. However, the second and third replicates were acquired soon after acquiring the 1st replicate, by taking the ORP probe out of the dispersion and reimmersing it back into the dispersion. The variations in the replicates here thus represent variations of the instrument's accuracy; they will not represent any variations that might be due to other factors such as differences in dispersion quality. Table 1 also shows the change in ORP values (values reported in brackets) upon addition of the nanomaterials relative to the corresponding blank media. Results show that, in most cases, there is a change of less than ~10 mV associated upon addition of the nanomaterials. There are only three cases in which the ORP value change is greater than 15 mV: Nanograin CeO_2_ in fish medium, Micron CeO_2_ in DI water, and Ceria dry CeO_2_ in fish media. Currently, we offer no explanation as to why there is a much larger change in ORP values in these three cases, apart from potential variations in dispersion quality, for example, due to potential redox contaminants associated with the different samples received. In addition, no real differentiation can be made between ZnO and CeO_2_ particles, with Z-COTE ZnO (BASF, Germany) having the same 9 mV change as the Ceria dry CeO_2_ (Antaria, Australia), when both are dispersed in DI water.

The other physicochemical characterization associated with the nanomaterials are shown in Tables 2, 3, and 4 below, which correspond to zetapotential, turbidity, and SEM particle size measurements, respectively. 

Results show that zetapotential values of nanomaterials when dispersed in seawater cannot be successfully measured (due to high conductivity) and thus displayed as N/A in Table 2. Such unsuccessful measurements were reported in the corresponding “quality report” at the end of the measurement. In general, results indicate high zetapotential values for nanomaterials that are dispersed either in DI water or DI water + 5 mM NaCl. Results of the DI water are similar to the corresponding DI water + NaCl case; the addition of NaCl into the DI water was carried out so as to have greater confidence in the DI water results, as the measurement of zetapotential usually involves the presence of inert background electrolyte. Overall, results show that nanomaterials are most stable when dispersed in DI water (or DI water + NaCl) and least stable when in an ecotox media. This is true apart for the case of Micron CeO_2_ showing that it is least stable in DI water, that is, −7 mV when compared to other ecotox media such as fish medium, that is, −22 mV. Currently, no explanation is available for this behaviour, and dispersion stability was further measured by using the concept of half-life values (as previously discussed in the introduction, the larger the half-life, the more stable the dispersion). Results in Table 3 show that, as with zetapotential measurements, results in DI water are generally more stable (with the largest associated with Z-COTE ZnO of 4038 minutes) than when in ecotox media. However, unlike the zetapotential values, Micron CeO_2_ also shows the same trend, that is, most stable in DI water than when in an ecotox media. The reason for this discrepancy lies in the fact that dispersion stability was measured in two different ways: through the measurement of interparticle force (zetapotential) or through analyzing the stability via sedimentation measurements (turbidity with time). The former measurement is solely governed by the electric properties of the solid surface in contact with liquid, which will subsequently contribute towards sedimentation rate; the latter measurement is not only determined by the zetapotential value but also by other factors, for example, particle size (in which the larger particles are expected to sediment at a much faster rate) [17]. The SEM results (Table 4) show the mean (Feret) primary particle sizes and the corresponding standard deviations (to reflect on the polydispersity of the primary particle size). Results presented in Table 4 show that the mean primary particle sizes of the samples range from ~30 nm to ~890 nm, the largest being the Micron ZnO and Micron CeO_2_ from Sigma Aldrich. The SD values show the large degree of polydispersity associated with samples received: high polydispersity associated with Sigma Aldrich samples, less so with Nanosun ZnO, Microniser. The SEM micrographs indicated that all particles tested here were highly aggregated together into agglomerates of irregular shape. 

If there is any particle contribution towards the final ORP values, then, out of the two types of nanomaterials tested, we expected CeO_2_ to have a bigger contribution compared to ZnO. This is on the basis that CeO_2_ particle can act as a redox couple of Ce(IV)/Ce(III), which is not the case for ZnO. If CeO_2_ particles had contributed to the final ORP value, then we expect this to occur with Nanograin CeO_2_ (having the smallest particle size of ~30 nm, as shown in Table 4) and when dispersed in DI water, as this resulted in a highly stable dispersion (noted by its high dispersion stability value of 2676 min and high zeta potential value of 33 mV, as shown in Tables 3 and 2, resp.). A highly stable dispersion will mean sufficient time to allow particles to diffuse to the Pt electrode, thus interacting with the Pt electrode in order to contribute towards the final ORP reading. Hence, we expected the redox potential to be affected most by the Nanograin CeO_2_ in DI water and clearly this was not the case. As shown in Table 2, Nanograin CeO_2_ in DI water only resulted in an ORP value change of 11 mV compared to a change of 21 mV when the same particles were dispersed in fish medium. Overall, results suggest that the particles have minor effects on the final ORP readings. 

## 4. Conclusion

The study investigated the redox potential measurements, using ORP probe electrode, of different ZnO and CeO_2_ dispersions, in various liquid media. The variations in the ORP readings for the different dispersions could not be regarded as being highly significant and were mainly governed by the type of liquid media that the nanomaterials were dispersed in. This is not surprising, as ORP values are dominated by the amount of dissolved chemical species in the liquid media. Dispersions in seawater resulted in the lowest ORP values, suggesting that the media is *reducing* in nature. This was attributed to a much higher concentration of reducing agents such as sulphites or a reduction in the concentration of dissolved oxygen under a high salinity environment. The study shows that there was little contribution from the particles themselves towards the final ORP reading, with no significant differentiation between CeO_2_ and ZnO. As it is clear that redox potential measurements using an ORP electrode will not indicate a particle's contribution towards the final redox potential value, the work has highlighted the need to have better tools for such measurements. There are several alternatives to using the ORP probe, including X-ray photoelectron spectroscopy (XPS) and electron paramagnetic resonance (EPR) and electron energy loss spectroscopy (EELS) [18]. However, these technologies rely on the indirect measurement of redox potential and the accuracy of the values reported may come into question. 

## Figures and Tables

**Table 1:** Redox potential of nanomaterial dispersion in various liquid media; the value quoted (in mV) is relative to the standard hydrogen reference electrode. The values in bracket show the difference in value with respect to the corresponding blank liquid media.

Sample name	Redox potential (mV) of nanomaterial dispersions			
	DI water	Fish medium	Seawater	Daphnia medium
Liquid media with no nanomaterials	405	418	384	425
Nanograin CeO_2_ (Umicore Belgium)	416 (11 mV)	439 (21 mV)	384 (0 mV)	415 (−10 mV)
Nanosun ZnO (Micronisers, Australia)	398 (−7 mV)	424 (6 mV)	380 (−4 mV)	415 (−10 mV)
Micron ZnO (Sigma Aldrich, UK)	398 (−7 mV)	430 (12 mV)	374 (−10 mV)	415 (−10 mV)
Z-COTE ZnO (BASF, Germany)	396 (−9 mV)	427 (9 mV)	379 (−5 mV)	422 (−3 mV)
Micron CeO_2_ (Sigma Aldrich, UK)	422 (17 mV)	430 (12 mV)	382 (−2 mV)	429 (4 mV)
Ceria dry CeO_2_ (Antaria, Australia)	414 (9 mV)	436 (18 mV)	387 (3 mV)	426 (1 mV)

**Table 2:** Mean values of zetapotential (of six replicates) for different nanomaterials dispersed in various media. DI water + 5 mM NaCl—this medium was employed to compare with the DI results when in the presence of inert background electrolyte. Values are the mean and ±1 SD of six replicates.

Sample name	DI water (mV)	DI water + 5 mM NaCl (mV)	Fish medium (mV)	Seawater (mV)	Daphnia medium (mV)
Nanograin CeO_2_	33.0 ± 2.0	33.9 ± 1.7	−11.1 ± 1.0	N/A	1.2 ± 0.2
Nanosun ZnO	24.6 ± 0.4	25.2 ± 0.6	12.4 ± 0.3	N/A	4.9 ± 0.2
Micron ZnO	20.2 ± 0.4	13.9 ± 0.6	4.4 ± 0.4	N/A	−4.6 ± 0.4
Z-COTE ZnO	24.3 ± 0.4	20.8 ± 0.8	10.8 ± 0.1	N/A	1.3 ± 0.2
Micron CeO_2_	−7.0 ± 6.0	−2.0 ± 2.0	−22.3 ± 0.5	N/A	−15.0 ± 0.3
Ceria Dry CeO_2_	28.0 ± 2.0	23.0 ± 1.3	−15.3 ± 0.6	N/A	−17.4 ± 0.3

**Table 3:** Dispersion stabilities, as measured by their corresponding “half-lives” (the time it takes for particle concentration to be reduced by half) of the different nanomaterials when dispersed in various media.

Sample name	DI water (min)	Fish media (min)	Seawater (min)	Daphnia media (min)
Nanograin CeO_2_	2676	282	288	252
Nanosun ZnO	2526	498	402	444
Micron ZnO	966	216	228	324
Z-COTE ZnO	4038	816	738	768
Micron CeO_2_	432	348	294	294
Ceria Dry CeO_2_	780	438	534	600

**Table 4:** The size of primary particles (of the “as-received” powders), as defined by their corresponding Feret's diameter. Mean diameter (±1 SD) of a minimum of 50–100 particles measured in the SEM images; the SD here represents the broadness of the size distribution.

Sample name	Supplier	Mean Feret diameter (±1 SD) from SEM images
Nanograin CeO_2_	Umicore Belgium	28.4 ± 10.4
Nanosun ZnO	Micronisers, Australia	42.5 ± 3.6
Micron ZnO	Sigma Aldrich, UK	891.8 ± 800.0
Z-COTE ZnO	BASF, Germany	151.0 ± 55.6
Micron CeO_2_	Sigma Aldrich, UK	615.3 ± 430.5
Ceria dry CeO_2_	Antaria, Australia	44.9 ± 14.6
